# Combination of intramuscular alfaxalone, butorphanol, and midazolam as a novel immobilization protocol in 3 ring-tailed lemurs (*Lemur catta*)

**DOI:** 10.1186/s13620-020-00163-1

**Published:** 2020-06-16

**Authors:** Kyratsoula Pentsou, Vilhelmiina Huuskonen

**Affiliations:** UCD Veterinary Hospital, School of Veterinary Medicine, University College Dublin, Belfield, Dublin 4 Ireland

**Keywords:** Ring-tailed lemur, *Lemur catta*, Alfaxalone, Butorphanol, Midazolam, Immobilization

## Abstract

**Background:**

There is very little data on the optimal anaesthetic management of ring-tailed lemurs, and the available information is mostly based on extrapolation from other species. In addition, a thorough pre-anaesthetic assessment of lemurs might not be possible without prior chemical immobilization, making a safe immobilization protocol essential.

**Case presentation:**

Three ring-tailed lemurs (*Lemur catta*) were immobilized using a combination of intramuscular alfaxalone (5 mg/kg), butorphanol (0.2 mg/kg), and midazolam (0.2 mg/kg), at the University College Dublin Veterinary Hospital. One lemur was anaesthetised once, two lemurs twice, amounting to five anaesthetic events. Conversion to general anaesthesia was warranted in all five occasions, and anaesthesia was maintained with either sevoflurane in oxygen or alfaxalone infusion. The immobilization protocol provided an adequate duration of deep sedation for diagnostic procedures and in some occasions allowed the intubation of the trachea. Analgesia was also provided for minor procedures. No major complications were noted with the protocol used.

**Conclusions:**

The combination of intramuscular alfaxalone, butorphanol and midazolam provided a clinically useful sedation/immobilization in ring-tailed lemurs with only minor complications such as mild hypothermia, hypotension, hypoventilation and bradycardia. This protocol could be considered in ring-tailed lemurs that need to be immobilized for minor procedures, or as a pre-anaesthetic premedication, especially if a full pre-anaesthetic clinical exam is not possible.

## Background

Ring-tailed lemurs belong to the family of Lemuriformes, a group of prosimian primates. They originate from the island of Madagascar but can be found all over the world in zoo facilities or as pets [1, 2]. However, there is not much data on their optimal anaesthetic management [3], and often pre-anaesthetic assessment might not be possible without prior chemical immobilization [2, 4]. Several injectable agents, including xylazine, medetomidine, dexmedetomidine, ketamine, tiletamine-zolazepam, diazepam, midazolam, and butorphanol [5, 6] have been used to sedate and/or immobilize prosimians. Out of these, ketamine is perhaps most commonly used [1, 2, 7], although the duration or degree of sedation is not consistent, and the quality of recovery is often poor [2]. Alpha_2_-agonists are also a popular component of sedation/immobilization protocols but cardiovascular and respiratory depression [3, 6], as well as thermoregulatory impairment [5] are potential adverse effects [7]. This report describes five occasions of intramuscular administration of a combination of alfaxalone, butorphanol and midazolam to three ring-tailed lemurs.

### Description of the cases

Three lemurs presented to our Veterinary Hospital for diagnostic investigations and surgery. One lemur was anaesthetised once, two lemurs twice, amounting to five anaesthetic events. All lemurs were manually restrained, and following a brief clinical examination, sedated with alfaxalone^1^ (5 mg/kg), butorphanol^2^ (0.2 mg/kg) and midazolam^3^ (0.2 mg/kg) mixed in the same syringe, and injected intramuscularly (IM) in the quadriceps muscle. No pain was noticed during the injection in any of the patients. Two to four minutes post-administration, sedation was deemed profound in all five occasions, and an intravenous (IV) catheter was aseptically placed in an external saphenous vein. Patients were placed in a prone position on a heated pad and administered 100% oxygen with a tight-fitting mask via a zero dead-space system,^4^ while physiologic parameters were monitored continuously and recorded every 5 min. In all five cases, general anaesthesia was necessary for further interventions. In two instances, IV administration of alfaxalone was needed to allow intubation, while in one case the patient was mask-induced with sevoflurane.^5^ The trachea was intubated with a cuffed or uncuffed endotracheal tube (ETT) following the installation of 2% lidocaine^6^ on the larynx. Anaesthesia was maintained with sevoflurane in 100% oxygen throughout, except for one case in which anaesthesia was maintained with alfaxalone infusion for the main part of the procedure. All lemurs received supportive intraoperative fluid therapy with a balanced crystalloid solution (Hartmann’s solution)^7^ at 10 ml/kg/h. Depth of anaesthesia was monitored by checking palpebral reflex, eye position, jaw tone and response to painful stimuli. Physiologic parameters such as end-tidal carbon dioxide (EtCO_2_)^8^^,^^9^ haemoglobin saturation (SpO_2_)^8,9^ end-tidal anaesthetic agent concentration (EtSevo)^9^, heart rate (HR)^8,9^ and rhythm^8,9^, respiratory rate (RR)^8,9^, and arterial blood pressure (either non-invasively using the oscillometric method^10^ or invasively^9,^^11^) were monitored continuously throughout the anaesthetic event and recorded every 5 min. Intermittent positive pressure ventilation^12^ was initiated in one case due to hypoventilation. Rectal temperature was measured every 15 min. In four out of five anaesthetic occasions multimodal analgesia was provided. Both local (epidural morphine) and systemic (fentanyl infusion, ketamine infusion, additional butorphanol, paracetamol and meloxicam) techniques were incorporated in order to provide intraoperative and postoperative pain relief. All three patients recovered smoothly from all anaesthetics. However, in two occasions midazolam reversal with intramuscular flumazenil^13^ (10 μg/kg) was opted to shorten the duration of recovery.

Additional information about the onset of sedation, requirement of additional anaesthetic agents, duration of general anaesthesia and intraoperative complications are summarized in Table 1.

**Table 1:** Additional information about the onset of sedation, requirement of additional anaesthetic agents, duration of general anaesthesia and intraoperative complications in three ring-tailed lemurs (*Lemur catta*)

	Anaesthetic 1	Anaesthetic 2	Anaesthetic 3	Anaesthetic 4	Anaesthetic 5
**Procedure**	X-rays; degloving injury repair; post-operative x-rays	CT; orthopaedic exam; tail amputation	Left foreleg amputation; tail amputation	CT	X-rays; left nephrectomy
**Onset of deep sedation (min)**	3	3	3	2	4
**Top-up for intubation**	Alfaxalone 1.5 mg/kg	None	None	Sevoflurane by mask	Alfaxalone 3 mg/kg
**Time elapsed between sedation and intubation (min)**	44	58	25	5	77
**EtSevo (%)**	Unknown	1.18–1.43	0.77–2.3	1.0–2.8	1.2–1.7
**Duration of general anaesthesia (min)**	66	34	171	55	146
**Time from end of anaesthesia to extubation (min)**	6	21	9	22	16
**Complications**	Hypothermia	Hypotension, hypothermia	None	Hypotension; hypothermia; bradycardia	Hypothermia; bradycardia; mild hypercarbia

### Case 1

In Spring 2019 a 4-year-old and 2.5 kg male ring-tailed lemur was presented for investigation of a degloving injury of the right thumb. Initial attempt to treat the wound had resulted in the animal licking the antibiotic ointment, so further diagnosis and treatment were considered necessary. During the physical examination, no signs of systemic disease [8] were identified. The alfaxalone, butorphanol and midazolam combination was administered intramuscularly, and the sedation had a profound effect within 3 min. While the patient was positioned for radiographic investigation of the affected limb, oxygen was supplemented, and SpO_2_, HR and rhythm, RR, and non-invasive arterial blood pressure (NIBP) and temperature (T) were monitored. Radiography revealed a luxation of the carpometacarpal joint of the first digit without fracture. Complete exposure of the subcutaneous tissue was present in the dorsal and partially in the palmar aspect of the right thumb. The animal was then moved to an operating room where its trachea was intubated after local installation of lidocaine 2% (2 mg/kg) on the larynx while an additional dose of alfaxalone (1.5 mg/kg IV) was administered (44 min after sedation), to allow the intubation with a 3.0 mm internal diameter (ID) uncuffed ETT. Immediately after, the patient was connected to a mini-Lack breathing system^14^ and general anaesthesia with sevoflurane initiated, while the patient was allowed to breathe spontaneously. After the surgical wound repair, repositioning of the luxated metacarpal bone and postoperative radiographs, sevoflurane was turned off and meloxicam^15^ was administered (0.1 mg/kg IV) for postoperative analgesia. The recovery was smooth and uneventful. Due to moderate residual sedation attributed to midazolam, the antagonist flumazenil was administered. The only complication was mild hypothermia (36.1 °C) [8].

### Case 2

In Spring 2019, another male lemur, 4 years old and weighing 2 kg, presented for investigation of suspected traumatic injury. Physical examination was within normal limits [8], while an orthopaedic examination confirmed paralysis of the left forelimb and necrosis of the proximal half of the tail. The alfaxalone, butorphanol and midazolam combination was administered intramuscularly and 3 min later sedation was profound. Computed tomography (CT) scan was performed to evaluate the severity of the damages and rule out any concurrent injuries. While positioned for the CT scan, oxygen was supplemented, and the animal was monitored as previously described. The animal was then prepared for surgical amputation of the necrotic proximal half of the tail, while amputation of the left antebrachium was planned for the following week. The trachea was intubated with a 3.0 mm ID cuffed ETT following installation of lidocaine (1 mg/kg) on the larynx, with no further intravenous anaesthetic drug administration required, 58 min after sedation. After intubation, the patient was connected to a mini-Lack breathing system and anaesthesia with sevoflurane initiated. The lemur was positioned in right lateral recumbency, covered with a forced warm air blanket,^16^ and was allowed to breathe spontaneously for the entire duration of the anaesthetic. Prior to the surgical incision, a ring block was performed proximally to the site of amputation with lidocaine (3 mg/kg). Due to hypotension (mean arterial pressure (MAP) 40 mmHg) and HR of 160 bpm, a bolus of glycopyrrolate^17^ (10 μg/kg IV) was administered with no result. Eventually, decreasing the sevoflurane concentration (EtSevo decreased from 1.43 to 1.18%) resolved the hypotension. Hypothermia (35.6 °C) was another intraoperative complication. After the surgery and postoperative radiography, anaesthesia was terminated and paracetamol^18^ (15 mg/kg IV) was administered for further postoperative analgesia. Flumazenil was administered and 2 min later the animal recovered smoothly and uneventfully. Paracetamol was continued (30 mg per os (PO)) three times daily (TID) for a week.

The same animal was presented again a week later for the left forelimb amputation. During the physical examination, some necrotic tissue was detected in the tail amputation site, and revision surgery was deemed necessary. The described sedation combination was administered, and 3 min later the patient was placed on a heated pad and oxygen was administered. Following lidocaine (1 mg/kg) splash on the larynx, the trachea was intubated with a 3.0 mm ID cuffed ETT, with no additional administration of intravenous anaesthetics (25 min after sedation), the patient connected to a mini-Lack breathing system and anaesthesia maintained with sevoflurane. During the forelimb amputation, additional analgesia was provided with ketamine^19^ infusion (5–10 μg/kg/min). Perineural infiltration of the brachial plexus with lidocaine (3 mg/kg) was performed prior to neurectomy, and a ring block with lidocaine (1 mg/kg) prior to tail amputation. Further analgesia was provided with butorphanol (0.2 mg/kg IV) and paracetamol (15 mg/kg IV). Recovery from anaesthesia was uneventful, and no intraoperative complications were detected. The animal was discharged with oral paracetamol.

### Case 3

In Summer 2019, a one-year-old female lemur, with a bodyweight of 1.56 kg, was presented for thoracic and abdominal CT scans due to intermittent anorexia and weight loss. Physical exam revealed no obvious abnormalities [8] other than body condition score of 2/9. The alfaxalone, butorphanol and midazolam combination was administered IM and profound sedation was observed 2 min after. For the CT scan, the patient was mask-induced with sevoflurane and the trachea was intubated with a 3 mm ID cuffed ETT after installation of lidocaine (3.8 mg/kg) on the larynx, 5 min after sedation. Immediately after the patient was connected to a paediatric T-piece breathing system^20^ and maintenance of anaesthesia with sevoflurane initiated while the patient was allowed to breathe spontaneously. After the CT scan, sevoflurane administration was stopped, and the recovery was smooth and rapid. Intraoperative complications included hypotension (MAP 42 mmHg) which responded to fluid boluses, bradycardia (120 bpm), and hypothermia (35.7 °C). CT findings showed an abnormal left kidney.

Two months later, the lemur presented again due to further weight loss, lethargy and pain. The same sedation protocol was used, and profound sedation was observed 4 min post-injection. Radiographs showed an irregular margination of the left kidney, and blood biochemistry analysis revealed marked azotaemia [9]. For the above reasons, a left ureteronephrectomy was planned. Tracheal intubation with a 3.5 mm ID cuffed ETT was achieved with an additional alfaxalone bolus (3 mg/kg IV), 75 min after sedation, and the patient was connected to a paediatric T-piece and received 100% oxygen while initially spontaneously ventilating. Total intravenous anaesthesia was maintained with alfaxalone infusion (0.06–2 mg/kg/min) and additional analgesia was provided with IV fentanyl^21^ infusion (2.6–8.4 μg/kg/h), epidural morphine^22^ (0.1 mg/kg), and paracetamol (10 mg/kg IV). For monitoring of arterial blood pressure, a 24G arterial catheter was placed in the coccygeal artery. Due to intraoperative hypercapnia, mechanical ventilation was started. Both IV infusions were terminated 30 min before the end of surgery and anaesthesia was maintained with sevoflurane for the remainder of the procedure to ensure a fast recovery. Recovery from anaesthesia was smooth and uneventful. Intraoperative complications included hypothermia (34.1 °C), bradycardia (110 bpm) and mild hypercapnia (EtCO_2_ 6.9 kPa). The patient was hospitalized overnight for monitoring and pain assessment. Postoperative analgesia consisted of IV paracetamol (10 mg/kg) TID; no additional analgesia was needed as the patient seemed comfortable. The animal was discharged the following morning with oral paracetamol (10 mg/kg, TID). Histopathologic examination of the left kidney was consistent with nephroblastoma (Wilm’s tumour), a congenital neoplasm that has been previously reported in other primates [10]. In the follow-up examination 1 week later, the lemur was interacting normally with its environment with no obvious signs of discomfort.

## Discussion

Concurrent and occult disease states can affect the safety of anaesthesia [2], but in wildlife species, pre-anaesthetic assessment might not always be possible in order to fully evaluate the health status [1, 11]. Even though ring-tailed lemurs can be manually restrained, allowing limited physical examination, for most procedures chemical immobilization is still required. However, there is very little data available [11] on the use of sedative drugs for lemurs [2, 3]. Most literature available is predominantly derived from the authors’ own experiences or extrapolated from other species [2, 11]. Thus, drugs and doses provided in the literature might not be the most appropriate for lemurs, and there is a need for evidence-based data. Several injectable drugs and their combinations have been used in the past in order to sedate/immobilize prosimians. The combinations described in the literature include medetomidine and ketamine [3], medetomidine with butorphanol and ketamine [3], medetomidine with butorphanol and midazolam [3], tiletamine-zolazepam and butorphanol [5], tiletamine-zolazepam with medetomidine and butorphanol [6], and dexmedetomidine with butorphanol and midazolam [7]. In addition, there are protocols involving only the use of a halogenated anaesthetic agent such as sevoflurane [12]. The most commonly described immobilization protocols for ring-tailed lemurs involve the use of a dissociative injectable anaesthetic such as ketamine or tiletamine [5], an alpha_2_-adrenergic agonist [4, 7], or the use of an inhalant agent [12].

Ketamine is a widely used drug for chemical restraint in primates [1]. The effect of ketamine in prosimians, however, is variable with the degree and duration of immobilization being unpredictable and the recovery period often accompanied by adverse reactions such as vomiting or seizures [2]. Ketamine has also been associated with poor muscle relaxation [4], and even muscle damage in primates [13]. Similarly, in a 2011 study [5], tiletamine and zolazepam did not provide adequate depth of anaesthesia or duration of immobilization for the desired procedures. Furthermore, both ketamine and tiletamine are known to cause pain during intramuscular injection [14], as well as prolonged recoveries. Last but not least, these anaesthetic agents can make tracheal intubation challenging since they do not completely abolish the swallowing reflex [5].

There is a query of whether or not alpha_2_-adrenergic agonists affect the cardiovascular system of ring-tailed lemurs in the same manner as in cats and dogs [3]. A study performed in 2003 [3] suggested that the previously described bradycardia in lemurs after alpha_2_-adrenergic agonist administration might depend on the route of administration and that intramuscular administration could possibly prevent it. Another study in 2011 [5], suggested that in ring-tailed lemurs medetomidine administration leads to bradycardia, decrease in ventilation and thermoregulatory compromise. Moreover, the alpha_2_-adrenergic agonists are often antagonized with atipamezole in order to shorten the duration of recovery, but there is good evidence that the antagonism not only reverses the sedative effects of the alpha_2_-adrenergic agonists but may also reverse the analgesia provided with opioids at the spinal level by blocking the non-adrenergic feedback inhibition of pain [15]. This is obviously not ideal if a painful procedure has been performed for which an opioid has been administered.

Similarly, mask-induction with an inhalant agent is often found in literature as a method to immobilize ring-tailed lemurs, even though, especially when used as a sole agent without prior sedation, high concentrations are required to anaesthetize the patient leading to profound cardiopulmonary depression [2, 16]. The administration of inhalant agent alone does not provide analgesia, and respiratory arrest has been observed in some lemur species that were mask-induced with isoflurane [2].

Our protocol for ring-tailed lemurs, which combines the intramuscular administration of alfaxalone (5 mg/kg), butorphanol (0.2 mg/kg) and midazolam (0.2 mg/kg), was designed in order to perform diagnostic procedures of unknown duration. In our hospital the same drug combination is often used to sedate fractious cats with a desirable outcome and lack of major complications. In this case report, our aim was to provide adequate sedation with minimal adverse effects, with the possibility to convert to general anaesthesia if needed. Furthermore, we needed a protocol that could be used in compromised patients in which a full pre-anaesthetic clinical exam was not possible. To our knowledge, the particular combination of drugs used in this case report for immobilization has not been previously described in ring-tailed lemurs.

Alfaxalone is a short-acting, non-irritant anaesthetic agent that can be administered via the intravenous or intramuscular route in many species without causing pain on injection [17]. In cats and dogs it provides good muscle relaxation and good cardiovascular and respiratory stability in clinically relevant doses [18, 19]. Alfaxalone is a versatile agent that can be used as a sedative, as an induction agent or in order to maintain general anaesthesia through constant rate administration. Only one study has reported its use in common marmosets (*Callithrix jacchus*) as a sedative agent but it was used alone and not in combination with other agents [4]. In another study, alfaxalone was used for maintenance of anaesthesia with continuous infusion in ring-tailed lemurs [7]. To our knowledge, the use of alfaxalone alone or in combination for immobilization in ring-tailed lemurs has not been described. Although the alfaxalone dose used resulted in a relatively high volume, none of the lemurs reacted during the IM injection.

Butorphanol is a synthetic opioid with mixed agonist-antagonist effects in most domestic species [20]. Specifically, it has agonist activity at kappa opioid receptors, while at mu-opioid receptors it acts as an antagonist [21] in most domestic species; it is mainly used for its sedative effects in companion animals as it only provides mild analgesia [22]. However, a 1995 study [20] demonstrated that in non-human primates butorphanol acts as a mu agonist with intermediate analgesic efficacy, rather than as a weak agonist-antagonist as in other species. The same study described a duration of action of up to 4 h in non-human primates [20], making butorphanol a commonly used opioid in ring-tailed lemurs [2]. In ring-tailed lemurs butorphanol has been observed to cause lower than normal respiratory rates but no obvious respiratory depression [3].

Midazolam is a benzodiazepine sedative that is commonly used in primates as a premedicant since it has minimal cardiorespiratory depressant effects [16], it can be administered intramuscularly, and its sedative effects can be antagonized with flumazenil if needed. In ring-tailed lemurs it provides anxiolysis, sedation, and muscle relaxation, and it has an anticonvulsant effect [2].

In all five events described in this case report, the sedation was deemed profound 2 to 4 min post-administration. In two occasions, the patients required additional alfaxalone (1.5 and 3 mg/kg IV) to enable tracheal intubation (44 and 77 min after sedation), while in one occasion, the patient was mask-induced with sevoflurane 5 min after sedation. In two occasions, endotracheal intubation was achieved without any additional drugs 25 and 58 min after IM sedation. The fact, that in two occasions additional injectable anaesthetic administration was required, was likely due to the time elapsed between the sedation administration and tracheal intubation. In Case 3, tracheal intubation was not attempted prior to the mask-induction with sevoflurane.

No major complications were noted with the use of the alfaxalone–butorphanol–midazolam combination. In two occasions IM flumazenil (10 μg/kg) was administered postoperatively in order to facilitate recovery, but it is unclear whether this was truly necessary as the duration of midazolam in primates is estimated to be around 20–30 min [2], and the time elapsed between midazolam and flumazenil was 120 and 140 min. In two occasions hypotension (MAP < 60 mmHg), in four occasions hypothermia (rectal temperature < 36.2 °C), and in two occasions bradycardia (HR < 145 bpm) was seen. The hypotension and bradycardia occurred only after induction of general anaesthesia, while hypothermia started to develop already during sedation for diagnostic procedures, worsening further during anaesthetic maintenance. The EtSevo concentrations measured in this case report were mostly lower (0.78–2.8%) than the previously published sevoflurane minimum alveolar concentration (MAC) values in ring-tailed lemurs (3.48 ± 0.55%) [12]. Even so, in both occasions in which hypotension was seen, the EtSevo concentrations were probably higher than needed to maintain adequate anaesthetic level, and the observed hypotension was the result of sevoflurane-induced dose-dependent vasodilation, rather than an effect of the drugs used for the sedation protocol. Therefore, it is likely that the drugs used in the sedation protocol had substantial anaesthetic-sparing effects. Very small animals are at a risk of anaesthesia-related hypothermia due to their large surface area to volume ratio [23]. Despite active warming throughout, four out of five lemurs became hypothermic, and we need to consider more aggressive heating methods in the future. Twice in the same lemur (Case 3) intraoperative bradycardia was noted. Potential causes include hypothermia [23], unnecessarily deep plane of anaesthesia, the concurrent renal neoplastic disease, or decreased sympathetic tone caused by butorphanol or fentanyl [21].

The analgesia provided by butorphanol was deemed adequate in order to perform diagnostic and minor surgical procedures; however, additional analgesia was considered necessary for amputations and laparotomy.

Unfortunately, this case report presents only a small number of animals. Additionally, most animals were not hospitalized following the anaesthetic event, and as a result, we might have missed some delayed postoperative complications. However, no postoperative complications were reported by the owners.

In conclusion, the combination of intramuscular alfaxalone (5 mg/kg), butorphanol (0.2 mg/kg) and midazolam (0.2 mg/kg) provided a clinically useful and reliable immobilization in ring-tailed lemurs with only minor intra- or postoperative complications. This protocol could be considered in ring-tailed lemurs that need to be immobilized for minor procedures, especially if a full pre-anaesthetic clinical exam is not possible.

## Data Availability

The data used are available from the corresponding author on reasonable request.

## Abbreviations

CT: Computed tomography
EtCO_2_: End-tidal carbon dioxide
EtSevo: End-tidal sevoflurane concentration
ETT: Endotracheal tube
HR: Heart rate
ID: Internal diameter
IM: Intramuscular
IV: Intravenous
MAP: Mean arterial pressure
NIBP: Non-invasive blood pressure
PO: Per os
RR: Respiratory rate
SpO_2_: Haemoglobin saturation with oxygen
T: Temperature
TID: Ter in die

## Footnotes

1 Alfaxalone: Alfaxan®, Jurox (Ireland) Limited

2 Butorphanol: Torbugesic^®^, Zoetis Belgium S.A

3 Midazolam: Hypnovel^®^, Roche Products Ltd., Welwyn Garden City, UK

4 Zero dead-space system: Non-Rebreathing ZDS Mask, Darvall, DarvallVet, Australia

5 Sevoflurane: Sevoflo®^,^ Abbott Animal Health, Dublin, Ireland

6 Lidocaine hydrochloride: Lidocaine hydrochloride 2%, B. Braun Medical Ltd., Dublin, Ireland

7 Hartmann’s solution: Vetivex 11^®^ (Hartmann’s) solution for infusion, Dechra Regulatory B.V

8 Mindray patient monitor: PM-9000 Express Patient monitor, Mindray, China

9 GE Healthcare patient monitor: B40 Patient monitor, GE, United States

10 Cardell 9401 Non-invasive Blood Pressure and Pulse Rate Veterinary Monitor, MIDMARK, U.S.A.

11 Blood pressure transducer: Medex single channel set invasive blood pressure transducer, Henleys Medical Supplies, UK

12 Small Animal Ventilator: SAV03 Small animal ventilator, Vetronic, Devon, UK

13 Flumazenil: Flumazenil, Fresenius, Kabi, Deuchland, GmbH

14 Mini-Lack breathing system: Mini-Lack Anaesthetic Breathing System, Burtons Medical Equipment, UK

15 Meloxicam: Loxicom, Morbrook Laboratories Limited, Newry, Co. Down, UK

16 Warming device: 3 M Bair Hugger® Warming Units, Canada

17 Glycopyrronium bromide: Glycopyrronium bromide, Mercury Pharmaceuticals Ltd.

18 Paracetamol: Paracetamol, Fresenius Kabi, Dublin, Ireland

19 Ketamine: Ketamidor, Richter Pharma, AG, Austria

20 Paediatric T-piece: Infant T-piece breathing system, Intersurgical Ltd., UK

21 Fentanyl: Sublimaze, Piramal Critical Care B.V., Netherlands

22 Morphine sulphate: Morphine sulphate, Mercury Pharmaceuticals (Ireland) Ltd.
