# Correction to: Combination of intramuscular alfaxalone, butorphanol, and midazolam as a novel immobilization protocol in 3 ring-tailed lemurs (*Lemur catta*)

**DOI:** 10.1186/s13620-020-00180-0

**Published:** 2020-12-05

**Authors:** Kyratsoula Pentsou, Vilhelmiina Huuskonen

**Affiliations:** UCD Veterinary Hospital, School of Veterinary Medicine, University College Dublin, Belfield, Dublin 4, Ireland

**Correction to: Ir Vet J (2020) 73:9**

**https://doi.org/10.1186/s13620-020-00163-1**

Following publication of the original article [1], it has been noticed that a reference was incorrect in a sentence.

The sentence currently reads:

However, there is not much data on their optimal anaesthetic management [3],

The sentence should read:

However, there is not much data on their optimal anaesthetic management [2],
